# Joint association of dietary index for gut microbiota and weekend warrior physical activity pattern with mortality among hypertensive patients

**DOI:** 10.1186/s12986-025-01026-8

**Published:** 2025-11-27

**Authors:** Jufeng Chen, Yuxi Chen, Sunanjie Zhao, Peiya Tao, Guohu Han, Zhuo Wang

**Affiliations:** 1 School of Medical and Health Engineering, Changzhou University, Changzhou, Jiangsu China; 2 Department of Nursing, Changzhou Second People’s Hospital, Affiliated Hospital of Nanjing Medical University, Changzhou, China

**Keywords:** Hypertension, Dietary index for gut microbiota, Weekend warrior, Mortality

## Abstract

**Background:**

The Dietary Index for Gut Microbiota (DI-GM) is a dietary quality indicator of gut health, weekend warrior (WW) has grown increasingly prevalent in today’s fast-paced lifestyle. The association of DI-GM and WW with mortality in hypertensive populations is currently unclear. This study aimed to explore the independent and joint associations of DI-GM and WW physical activity pattern with mortality among hypertensive patients.

**Methods:**

Utilizing data from 9082 hypertensive patients in the NHANES 2007–2018. The WW physical activity pattern was assessed using the self-reported Global Physical Activity Questionnaire, and DI-GM was estimated based on 24-hour dietary recalls. Kaplan–Meier (KM) curves and the Cox proportional hazard model were used to evaluate the associations between independent and joint prognostic effects of DI-GM and WW with mortality among hypertensive patients.

**Results:**

During a median follow-up of 6.8 years, 919 deaths were recorded. Analysis showed that DI-GM was associated with all-cause mortality in hypertensive patients (HR = 0.94, 95% CI [0.90, 0.99]). The all-cause (HR = 0.59, 95%CI 0.47 to 0.74) and cardiovascular disease (CVD) mortality (HR = 0.51, 95%CI 0.33 to 0.79) were significantly lower for weekend warriors compared with inactive individuals. The combined analysis demonstrated that, compared to individuals with low DI-GM and inactivity, those with high DI-GM and WW had a 55% lower risk of all-cause mortality, while those with low DI-GM and WW exhibited a 47% reduced risk of CVD mortality.

**Conclusions:**

DI-GM demonstrated a significant inverse association with all-cause mortality risk among hypertensive patients. WW had similar benefits for all-cause mortality as regularly active. Our study revealed that the combination of high DI-GM diets with WW was robustly associated with reduced all-cause mortality risk in hypertensive patients.

**Supplementary Information:**

The online version contains supplementary material available at 10.1186/s12986-025-01026-8.

## Introduction

 Hypertension is a prevalent chronic disease and a leading risk factor for cardiovascular and cerebrovascular events. According to the World Health Organization, there are currently over 1.3 billion individuals with hypertension worldwide [1]. Additionally, approximately 10 million people die from hypertension-related diseases annually [2], and this mortality rate continues to rise. A healthy diet and recommended levels of physical activity (PA) play a critical role in controlling hypertension and reducing the risk of its complications.

In recent years, the PA pattern of “weekend warrior” (WW) has garnered attention. The term “weekend warrior” describes individuals who, due to busy work schedules, engage in minimal PA during the weekdays but focus on high-intensity and prolonged exercise sessions over the weekends [3]. While moderate and consistent daily PA is well established to improve cardiovascular health and metabolic efficiency [4], the irregular rhythm of WW may elicit distinct physiological responses, particularly regarding muscle recovery, joint adaptation, and cardiovascular stress. Several studies in hypertensive adults have demonstrated that WW significantly reduces all-cause mortality and CVD mortality [5, 6]. One prior study has explored the associations between the different active patterns with all-cause mortality in individuals with hypertension as a subgroup analysis and found that, compared with physical inactivity, WW was associated with a lower risk of all-cause mortality [7]. WW may confer cardiometabolic benefits in hypertensive patients [8]. Nevertheless, Cao et al. [9] reported that the negative correlation between WW and hypertension was not statistically significant compared to inactivity. Dos Santos et al. [10] examined the association of leisure-time PA patterns with all-cause and cause-specific mortality in a large cohort of 350,978 adults in the US and also found a non-statistically significant association between WW with all-cause and CVD mortality. Despite these mixed findings, the underlying mechanisms of WW and its specific impact on hypertensive populations require further investigation.

Increasing evidence implicates gut microbiota as a mediator of the association between diet and human health. However, few dietary patterns to date have explicitly integrated the gut microbial profile. To address this gap, the Dietary Index of the Gut Microbiota (DI-GM), first proposed by Kase et al. [11], was developed as a literature-derived index to evaluate diet quality in relation to gut health. The validity of this scoring system has been demonstrated through biomarkers such as microbial diversity indices and short-chain fatty acids (SCFAs) production. At present, DI-GM has been applied to investigate the role of diet-induced gut microbiota disorders in the pathogenesis of various diseases [11]. A study reported a strong association between the DI-GM and a lower risk of hypertension [12]. Within the DI-GM construct, fermented dairy products represent an important food group because they are rich in live microorganisms that directly modulate the gut microbiota, enhance microbial diversity, and increase SCFA production [11, 13]. Evidence suggests that moderate intake of these live microbes is associated with a lower risk of CVD mortality compared with non-consumption, highlighting their biological relevance to the DI-GM construct [14]. Conversely, dietary patterns that promote sulfur-metabolizing gut bacteria have been linked to adverse health outcomes. For example, Deng et al. [15] reported that a sulfur-metabolizing diet, which supports the growth of bacteria producing harmful metabolites such as hydrogen sulfide, was associated with higher all-cause and cause-specific mortality in American adults. The DI-GM incorporates such negative dietary components by accounting for shifts in microbial composition and specific bacterial abundances, thereby capturing both protective and detrimental influences of diet on gut health. Despite this evidence, the association between the DI-GM and mortality in hypertensive populations has yet to be systematically examined.

Although the independent associations of PA and diet with mortality are apparent, much of the evidence base comes from studies evaluating PA or diet independently [16–18]. Results of a few studies that have assessed the combined association of diet and PA with health outcomes and mortality [19–24], revealed that sufficient vigorous leisure-time PA together with adherence to an anti-inflammatory diet was associated with better improvement in all-cause and CVD mortality compared to either behavior alteration alone [19], but only one study indicated a significant interaction between PA and diet [20]. Results were inconsistent; two studies [21, 22] indicated that high PA levels did not fully counteract the detrimental associations of a poor diet on mortality risk, while others [23, 24] demonstrated that even one healthy behaviour could lower all-cause mortality. These mixed results underscore the need to clarify the joint roles of PA and diet in mortality risk.

The gut microbiota is a potential pathway linking these behaviours to health outcomes. PA has been shown to increase gut microbial diversity and promote SCFA-producing taxa such as Roseburia and Faecalibacterium, thereby enhancing intestinal barrier function and reducing systemic inflammation [25]. Interestingly, Zhang et al. [26] found that short-term, high-intensity training (similar to WW) did not significantly alter SCFA levels in mice, suggesting WW may exert distinct gut microbial effects compared with regular PA. Together, these findings raise the hypothesis that WW or a high DI-GM diet may compensate for the absence of the other, highlighting the importance of examining their independent and joint associations.

A better understanding of the association between dietary intake and PA with mortality, especially in hypertensive patients who are not able to adhere to lifestyle changes in both nutrition and PA simultaneously, may help to identify appropriate and feasible strategies to reduce the burden of the diseases. Thus, we used data from the National Health and Nutrition Examination Survey (NHANES) to investigate the independent and interactive effects of DI-GM and WW on mortality among hypertensive patients.

## Methods

### Study population

**Fig. 1:**
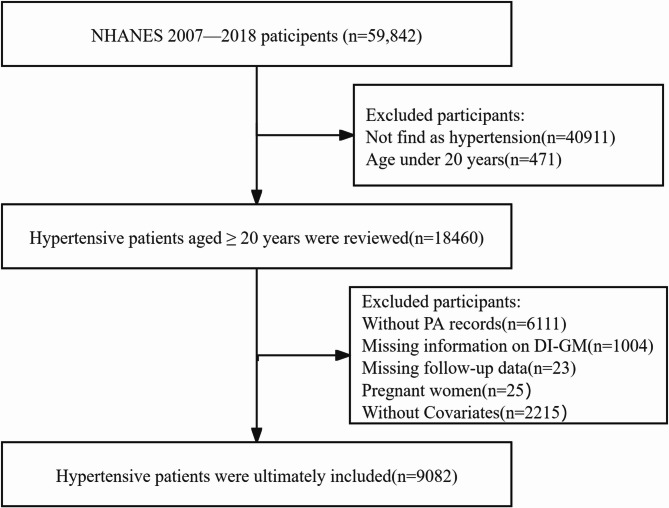
Flowchart of Participant Selection from NHANES 2007–2018

The study utilized data from NHANES, a comprehensive research program conducted by the Centres for Disease Control and Prevention aimed at assessing the health and nutritional status of the American population through interviews, examinations, dietary, and laboratory data. The NHANES protocol was approved by the Institutional Review Board of the National Centre for Health Statistics, adhering to the Declaration of Helsinki, with informed consent obtained from all participants. For this study, we focused on participants in the NHANES 2007–2018 cycle, as these cycles included the DI-GM assessment relevant to our study. Initially, a total of 59,842 participants were considered. After excluding individuals who did not have hypertension, were younger than 20 years of age, had no PA data, lacked DI-GM diet data, had no follow-up mortality data, were pregnant, and were missing covariates, our final analytic cohort consisted of 9082 hypertensive patients. (details provided in Fig. 1)

### Definition of hypertension

The diagnostic criteria for hypertension included mean SBP ≥ 130 mmHg, mean DBP ≥ 80 mmHg, ongoing antihypertensive therapy, or self-reported physician diagnosis of hypertension [27].

### Assessment of DI-GM

The DI-GM score is based on the intake of 14 specific foods or nutrients. Avocado, broccoli, chickpeas, coffee, cranberries, fermented dairy products, dietary fiber, green tea, soy, and whole grains are classified as beneficial components, while red meat, processed meat, refined grains, and high-fat diets (≥ 40% of energy from fat) are considered detrimental components [10]. For beneficial foods, participants with an intake exceeding the sex-specific median are assigned a value of 1, while those below the median receive a value of 0. In contrast, for unfavorable foods, an intake above the sex-specific median is assigned a value of 0 and a value of 1 is given to those below the median. A higher DI-GM score indicates a healthier gut microbiota. The total DI-GM score, ranging from 0 to 13 (with a beneficial range of 0–9 and a detrimental range of 0–4), was generated by summing all individual scores. In NHANES, the components of the DI-GM were derived from data obtained through two 24-h dietary recalls of all food and drink consumed on the day before the interview (from midnight to midnight), with a time interval varying from 3 to 10 days. The first dietary recall interview was collected face-to-face in the Mobile Examination Center (MEC) and the second interview was collected by telephone 3 to 10 days later. The mean intake of foods, food groups, and nutrients from the two 24-h recalls was used to construct the DI-GM. Previous studies have demonstrated that DI-GM score of ≥ 6 shows a significant negative correlation with adverse clinical outcomes [28, 29]. In the present study, the DI-GM scores were categorized into two groups: 0–5, and ≥ 6.

### Assessment of weekend warrior

PA was measured through the Global Physical Activity Questionnaire (GPAQ). The total minutes of moderate-intensity PA (MPA) and vigorous-intensity PA (VPA) were calculated by multiplying the weekly frequency by the duration per session. One minute of VPA was considered equivalent to 2 min of MPA [30]. PA frequency was defined as the maximum number of sessions per week of either MPA or VPA. Total weekly PA was computed using the following formula: total minutes of PA = (MPA frequency per week × duration per session) + 2 × (VPA frequency per week × duration per session). Based on total weekly PA duration, participants were categorized into three groups: inactive (total minutes of PA < 150 min per week), weekend warrior (total minutes of PA ≥ 150 min per week, performed in one or two sessions), and regularly active (total minutes of PA ≥ 150 min per week, distributed over more than two sessions). In addition, GPAQ allowed us to capture PA across different domains, including occupational PA and leisure-time PA. Both domains were incorporated into the calculation of total weekly PA.

### Determination of mortality outcomes

The main outcomes in the current study were the survival condition of participants, which was determined by referring to the National Death Index through 31 December 2019. All-cause mortality was defined as deaths attributable to any cause. In addition, CVD mortality was defined as deaths resulting from heart diseases (I00–I09, I11, I13, and I20–I51) and cerebrovascular diseases (I60–I69) based on the International Classification of Diseases, 10th Revision.

#### Covariates assessment

The selection of potential covariates was based on prior knowledge of factors that influence prognosis in patients with hypertension. In the study, the following covariates were included for descriptive and inferential analyses: (i) Sociodemographic characteristics—age (grouped as < 45, and ≥ 45 years), sex (male/female), race/ethnicity (Mexican American, non-Hispanic White, non-Hispanic Black, Other), family size (1–2, 3–4, ≥ 5), education (< 9th grade, high school or equivalent, college or above), and marital status (divorced/widowed/separated, married/living with partner and not married); (ii) Lifestyle and behavioral variables—smoking status (never, former, now), alcohol consumption (never, former, now), and dietary quality measured by the DASH score (treated as a continuous variable in primary analyses and categorized into tertiles/quartiles for sensitivity analyses); body mass index (BMI) was measured at the physical examination and modeled as a continuous variable; (iii) Health conditions and treatment—self-reported physician-diagnosed hyperlipidemia, diabetes mellitus, and cancer; sitting time (SBG) when available (categorized as < 240, 240–360, ≥ 360 min/day); and medication use including antidiabetic drugs, antihypertensive agents, and lipid-lowering therapies (each coded yes/no). Health insurance status (yes/no) was included to account for differences in healthcare access.

#### Statistical analysis

Baseline characteristics are summarized according to the DI-GM levels (high, low) and PA patterns (inactive, weekend warrior, regularly active). Continuous variables are presented as mean ± standard error (SE) or median (interquartile range), and categorical variables are shown as numbers (percentage, %). Continuous variables with normal distribution were evaluated using Student’s t-test, while continuous variables with non-normal distribution were tested using the Kruskal–Wallis test. Categorical variables were compared by using the χ^2^ test.

Restricted cubic spline (RCS) regression was employed to flexibly model the association of DI-GM scores and PA time with mortality risk. We applied a weighted multivariable Cox proportional hazards model to test the associations of DI-GM levels and PA patterns with hypertensive patients’ mortality. The proportional hazards assumptions were tested using Schoenfeld tests, and the P value was not significant in all models. To explore the joint exposure effects on the mortality of hypertensive patients, the participants were classified according to the DI-GM levels and PA patterns. We evaluated the interaction effects between DI-GM levels and PA patterns on the onset of mortality using measures on both multiplicative and additive scales. Furthermore, Kaplan–Meier (KM) curves were utilized to display the different survival probabilities among hypertensive patients with different DI-GM and PA phenotypes. Subgroup analyses were performed by age (< 60 vs. ≥60 years), sex (male vs. female), and sedentary time (< 6 vs. ≥6 h/day) to examine potential effect modification. Sensitivity analysis was conducted by excluding participants who died during the first 24 months of follow-up to minimize the potential impact of reverse causation.

Statistical analyses were performed with R version 4.4.2 (R Foundation for Statistical Computing, Vienna, Austria). All P-values were two-sided, and *P* < 0.05 was considered statistically significant.

## Results

### Baseline characteristics

A total of 9,082 hypertensive patients were ultimately included in this study (weighted population:60,440,371, weighted median age: 53 years, and weighted female proportion: 44.4%). Among the participants, 71.36% (*n* = 4,077) were non-Hispanic White; 62.80% (*n* = 4,995) had more than a college education; and 66.72% (*n* = 5,575) were married or living with a partner. At the time of the interview, over half of the study population were still drinking (*n* = 6,395), and 17.61% were current smokers (*n* = 1,698). The prevalence rates of comorbidities were as follows: diabetes mellitus 29.32% (*n* = 3,046), hyperlipidemia 78.02% (*n* = 6,998), and cancer 13.92% (*n* = 1,137). With 60.59% and 39.41% classified as low and high DI-GM, respectively. Participants with diets more conducive to gut health were likely to smoke less, have a lower BMI, have higher literacy levels, and drink less alcohol. Overall, 1,912 participants were inactive, 2,496 were WW, and 4,674 were regularly active. WW are likely to have a high school or equivalent degree, smoke less, spend less time sitting, and have higher levels of PA. The detailed characteristics of the population with varied levels of DI-GM and PA patterns are summarized in Table 1.

**Table 1:** Participants’ characteristics at baseline according to DI-GM levels and PA patterns in NHANES 2007–2018

Variable	Total	DI-GM group			Physical activity patterns			
		Low DI-GM	High DI-GM	*P* value	Inactive	Weekend warrior	Regularly active	*P* value
**Overall Participants**	9082	5503	3579		1912	2496	4674	
**Age**,** years**	54.00(42.00,65.00)	53.00(40.00,63.00)	57.00(46.00,67.00)	**< 0.0001**	57.00(46.00,68.00)	53.00(39.00,64.00)	54.00(42.00,64.00)	**< 0.0001**
**Age**				**< 0.0001**				**< 0.0001**
Age < 45 years	2405(28.64)	1659(32.71)	746(22.77)		363(21.53)	815(33.32)	1227(28.60)	
Age ≥ 45 years	6677(71.36)	3844(67.29)	2833(77.23)		1549(78.47)	1681(66.68)	3447(71.40)	
**Sex**				**0.01**				**< 0.0001**
Female	4009(44.40)	2338(42.47)	1671(47.17)		1075(57.84)	879(34.08)	2055(45.34)	
Male	5073(55.60)	3165(57.53)	1908(52.83)		837(42.16)	1617(65.92)	2619(54.66)	
**Race**				**< 0.0001**				**0.18**
Mexican American	1055(5.87)	672(6.52)	383(4.94)		214(5.32)	262(5.44)	579(6.34)	
Non-Hispanic black	2236(12.12)	1536(14.29)	700(9.01)		493(13.50)	626(11.43)	1117(12.00)	
Non-Hispanic white	4077(71.36)	2319(68.67)	1758(75.23)		846(70.50)	1145(72.41)	2086(71.06)	
Other race	1714(10.65)	976(10.52)	738(10.83)		359(10.67)	463(10.71)	892(10.60)	
**Education level**				**< 0.0001**				**< 0.0001**
Less than 9th grade	720(3.64)	467(4.02)	253(3.11)		182(4.65)	136(2.36)	402(4.02)	
High school or equivalent	3367(33.56)	2266(37.73)	1101(27.55)		727(33.73)	820(28.46)	1820(36.54)	
College or above	4995(62.80)	2770(58.26)	2225(69.34)		1003(61.62)	1540(69.18)	2452(59.44)	
**Marital status**				**0.003**				**0.01**
Divorced/widowed/separated	2278(20.72)	1384(20.63)	894(20.84)		578(24.31)	570(18.87)	1130(20.42)	
Married/living with partner	5575(66.72)	3290(65.47)	2285(68.51)		1115(65.00)	1540(67.07)	2920(67.17)	
Never married	1229(12.57)	829(13.90)	400(10.65)		219(10.69)	386(14.06)	624(12.41)	
**Insurance**				**< 0.0001**				**0.002**
No	1560(13.41)	1079(15.21)	481(10.82)		270(11.43)	398(12.08)	892(14.98)	
yes	7522(86.59)	4424(84.79)	3098(89.18)		1642(88.57)	2098(87.92)	3782(85.02)	
**Family size**,** people**				**< 0.0001**				0.58
1 ~ 2	5078(58.49)	2907(54.71)	2171(63.94)		1103(60.40)	1386(57.39)	2589(58.41)	
3 ~ 4	2579(29.43)	1616(31.44)	963(26.53)		531(27.71)	721(30.24)	1327(29.62)	
≥ 5	1425(12.08)	980(13.85)	445(9.54)		278(11.89)	389(12.38)	758(11.97)	
**smoke**				**< 0.0001**				**0.02**
Nerve	2652(30.53)	1516(28.99)	1136(32.73)		564(29.69)	737(31.74)	1351(30.12)	
Former	4732(51.87)	2796(51.21)	1936(52.81)		1013(52.59)	1318(53.38)	2401(50.67)	
Now	1698(17.61)	1191(19.80)	507(14.46)		335(17.71)	441(14.88)	922(19.20)	
**Drinking status**				0.3				**0.002**
Never	1158(9.48)	699(9.84)	459(8.96)		268(9.72)	253(7.50)	637(10.58)	
Former	1529(13.82)	946(14.19)	583(13.29)		400(16.29)	352(12.65)	777(13.56)	
Now	6395(76.70)	3858(75.97)	2537(77.75)		1244(73.99)	1891(79.85)	3260(75.86)	
**Sitting time**,** min/d**	360.00(240.00,480.00)	300.00(240.00,480.00)	360.00(240.00,480.00)	0.07	360.00(240.00,600.00)	360.00(240.00,480.00)	300.00(180.00,480.00)	**< 0.0001**
**Sitting time**,** min/d**				0.17				**< 0.0001**
< 240	2515(23.31)	1542(23.82)	973(22.58)		403(16.32)	700(23.86)	1412(25.72)	
240–359	3498(39.71)	1820(35.92)	1249(38.49)		857(49.17)	828(37.41)	1384(31.96)	
≥ 360	3069(36.97)	2141(40.25)	1357(38.93)		652(34.51)	968(38.73)	1878(42.33)	
**Chronic disease**								
**Diabetes Mellitus**				0.12				**< 0.0001**
No	6036(70.68)	3626(69.80)	2410(71.95)		1157(65.04)	1753(74.26)	3126(70.74)	
Yes	3046(29.32)	1877(30.20)	1169(28.05)		755(34.96)	743(25.74)	1548(29.26)	
**Hyperlipidemia**	2084(21.98)			0.27				**0.002**
No	6998(78.02)	1300(22.53)	784(21.19)		365(17.91)	604(23.05)	1115(22.93)	
Yes		4203(77.47)	2795(78.81)		1547(82.09)	1892(76.95)	3559(77.07)	
**Cancer**				**0.002**				0.4
No	7945(86.08)	4872(87.38)	3073(84.21)		1650(84.96)	2202(86.68)	4093(86.17)	
Yes	1137(13.92)	631(12.62)	506(15.79)		262(15.04)	294(13.32)	581(13.83)	
**Drug use**								
**Antidiabetic drugs**				0.08				**< 0.0001**
No	7704(87.68)	4657(86.96)	3047(88.72)		1542(83.93)	2177(90.62)	3985(87.38)	
Yes	1378(12.32)	846(13.04)	532(11.28)		370(16.07)	319(9.38)	689(12.62)	
**AntiHypertension drugs**				**0.002**				**< 0.0001**
No	4487(52.57)	2818(54.57)	1669(49.70)		774(43.69)	1345(57.51)	2368(53.08)	
Yes	4595(47.43)	2685(45.43)	1910(50.30)		1138(56.31)	1151(42.49)	2306(46.92)	
**AntiHyperlipidemic drugs**				0.11				**0.01**
No	6497(72.96)	4021(73.75)	2476(71.84)		1293(69.32)	1814(74.18)	3390(73.66)	
Yes	2585(27.04)	1482(26.25)	1103(28.16)		619(30.68)	682(25.82)	1284(26.34)	
**BMI**	29.30(25.70,33.90)	29.74(26.03,34.50)	28.80(25.40,33.20)	**< 0.0001**	30.00(26.00,35.40)	28.82(25.77,32.70)	29.40(25.68,34.00)	**< 0.0001**
**SBP**	130.00(120.00,140.00)	130.00(121.00,140.00)	130.00(120.00,140.00)	0.18	131.00(120.00,141.00)	129.00(120.00,139.00)	131.00(120.00,140.00)	**0.04**
**DBP**	77.00(68.00,84.00)	78.00(68.00,84.00)	76.00(67.00,83.00)	**< 0.0001**	76.00(66.00,82.00)	78.00(69.00,84.00)	77.00(68.00,84.00)	**< 0.001**
**DASH**	2.00(1.00,3.00)	1.50(1.00,2.50)	2.50(1.50,3.50)	**< 0.0001**	2.00(1.00,3.00)	2.00(1.00,3.00)	2.00(1.00,3.00)	0.46
**DI-GM**	5.00(4.00,6.00)	4.00(3.00,5.00)	7.00(6.00,7.00)	**< 0.0001**	5.00(4.00,6.00)	5.00(4.00,6.00)	5.00(4.00,6.00)	0.36
**Unfavorable to gut microbiota**	3.00(2.00,3.00)	2.00(1.00,3.00)	3.00(3.00,4.00)	**< 0.0001**	3.00(2.00,3.00)	3.00(2.00,3.00)	3.00(2.00,3.00)	0.06
**Beneficial to gut microbiota**	3.00(2.00,4.00)	2.00(1.00,3.00)	4.00(3.00,5.00)	**< 0.0001**	3.00(2.00,4.00)	3.00(2.00,4.00)	3.00(2.00,4.00)	0.09
**PAtime**	500.00(180.00,1360.00)	540.00(180.00,1500.00)	480.00(180.00,1200.00)	**0.003**	75.00(50.00, 120.00)	720.00(360.00,1485.00)	750.00(360.00,1800.00)	**< 0.0001**

### Association of DI-GM and PA patterns with all-cause and CVD mortality among hypertensive patients

During a median follow-up of 6.8 years, 919 deaths were documented, including 273 participants who died from CVD. As presented in Table 2, all-adjusted models showed a significant association between DI-GM and all-cause mortality in hypertensive patients (HR = 0.94, 95%CI: 0.90–0.99, *P* = 0.02). Notably, beneficial to gut microbiota were associated with a reduced risk of all-cause and CVD mortality(HR = 0.89, 95%CI: 0.83–0.95, *P <* 0.001; HR = 0.86, 95%CI: 0.76–0.96, *P* = 0.01); unfavorable to gut microbiota were not significantly associated with increased mortality(HR = 1.09, 95%CI: 0.99–1.20, *P =* 0.08; HR = 1.09, 95%CI: 0.94–1.26, *P* = 0.27). For WW and regularly active participants, they were associated with reduced all-cause mortality in hypertensive patients. Compared with physically inactive participants, the HRs for all-cause mortality were 0.59 (95%CI 0.47 to 0.74) for WW, and 0.76 (95%CI 0.63 to 0.91) for regularly active participants. When compared with physically inactive individuals, only WW were associated with a lower CVD mortality risk (HR = 0.51, 95%CI 0.33 to 0.79) (Table 3).

**Table 2:** Association between DI-GM and mortality among hypertensive patients in NHANES 2007–2018

Mortality outcome	Model 1		Model 2		Model 3	
	HR (95% CI)	*P*	HR (95% CI)	*P*	HR (95% CI)	*P*
All-cause mortality						
DI-GM	0.97(0.93,1.02)	0.26	0.94(0.90,0.99)	**0.02**	0.94(0.90,0.99)	**0.02**
Unfavorable to gut microbiota	1.12(1.02,1.23)	**0.02**	1.12(1.02,1.23)	**0.02**	1.09(0.99,1.20	0.08
Beneficial to gut microbiota	0.90(0.85,0.96)	**0.001**	0.86(0.81,0.92)	**< 0.0001**	0.89(0.83,0.95)	**< 0.001**
DI-GM						
0–3	Reference	Reference	Reference	Reference	Reference	Reference
4	0.88(0.65,1.18)	0.39	0.89(0.66,1.20)	0.45	0.88(0.66,1.17)	0.36
5	0.77(0.59,1.00)	**0.05**	0.74(0.57,0.97)	**0.03**	0.82(0.65,1.04)	0.1
≥ 6	0.83(0.66,1.05)	0.12	0.74(0.58,0.93)	**0.01**	0.74(0.59,0.93)	**0.01**
CVD mortality						
DI-GM	0.94(0.86,1.03)	0.18	0.91(0.82, 0.99)	**0.04**	0.92(0.84, 1.01)	0.09
Unfavorable to gut microbiota	1.07(0.93,1.24)	0.35	1.08(0.94, 1.25)	0.26	1.09(0.94, 1.26)	0.27
Beneficial to gut microbiota	0.88(0.79,0.98)	**0.02**	0.82(0.73, 0.92)	**< 0.001**	0.86(0.76, 0.96)	**0.01**
DI-GMQ						
0–3	Reference	Reference	Reference	Reference	Reference	Reference
4	0.64(0.41,0.98)	**0.04**	0.65(0.42, 1.01)	0.06	0.70(0.45, 1.09)	0.12
5	0.57(0.34,0.97)	**0.04**	0.55(0.32, 0.95)	**0.03**	0.66(0.39, 1.12)	0.12
≥ 6	0.66(0.45,0.95)	**0.02**	0.57(0.38, 0.84)	**0.004**	0.62(0.42, 0.93)	**0.02**

Model 1 served as the unadjusted analysis

Model 2: further adjusted for sociodemographic characteristics (age, sex, and race)

Model 3: further adjusted for personal lifestyle and behavioral variables (education level, family size, insurance, marital status, smoking status, drinking status, BMI, sitting time, DASH), factors associated with health conditions (diabetes mellitus, hyperlipidemia, cancer, antidiabetic drugs, antihypertensive drugs, and lipid-lowering therapies)

**Table 3:** Association between PA patterns and mortality in hypertensive patients in NHANES 2007–2018

Mortality outcome	Model 1		Model 2		Model 3	
	HR (95% CI)	*P*	HR (95% CI)	*P*	HR (95% CI)	*P*
All-cause mortality						
Inactive	Reference	Reference	Reference	Reference	Reference	Reference
Weekend warrior	0.52(0.42,0.66)	**< 0.0001**	0.52(0.41,0.66)	**< 0.0001**	0.59(0.47,0.74)	**< 0.0001**
Regularly active	0.74(0.62,0.88)	**< 0.001**	0.73(0.61,0.88)	**0.001**	0.76(0.63,0.91)	**0.003**
CVD mortality						
Inactive	Reference	Reference	Reference	Reference	Reference	Reference
Weekend warrior	0.47(0.31,0.73)	**< 0.001**	0.45(0.29, 0.69)	**< 0.001**	0.51(0.33, 0.79)	**0.002**
Regularly active	0.70(0.50,0.99)	**0.04**	0.67(0.48, 0.95)	**0.02**	0.71(0.50, 1.00)	**0.05**

Model 1 served as the unadjusted analysis;

Model 2: further adjusted for sociodemographic characteristics (age, sex, and race);

Model 3: further adjusted for personal lifestyle and behavioral variables (education level, family size, insurance, marital status, smoking status, drinking status, BMI, sitting time, DASH), factors associated with health conditions (diabetes mellitus, hyperlipidemia, cancer, antidiabetic drugs, antihypertensive drugs, and lipid-lowering therapies)

**Fig. 2:**
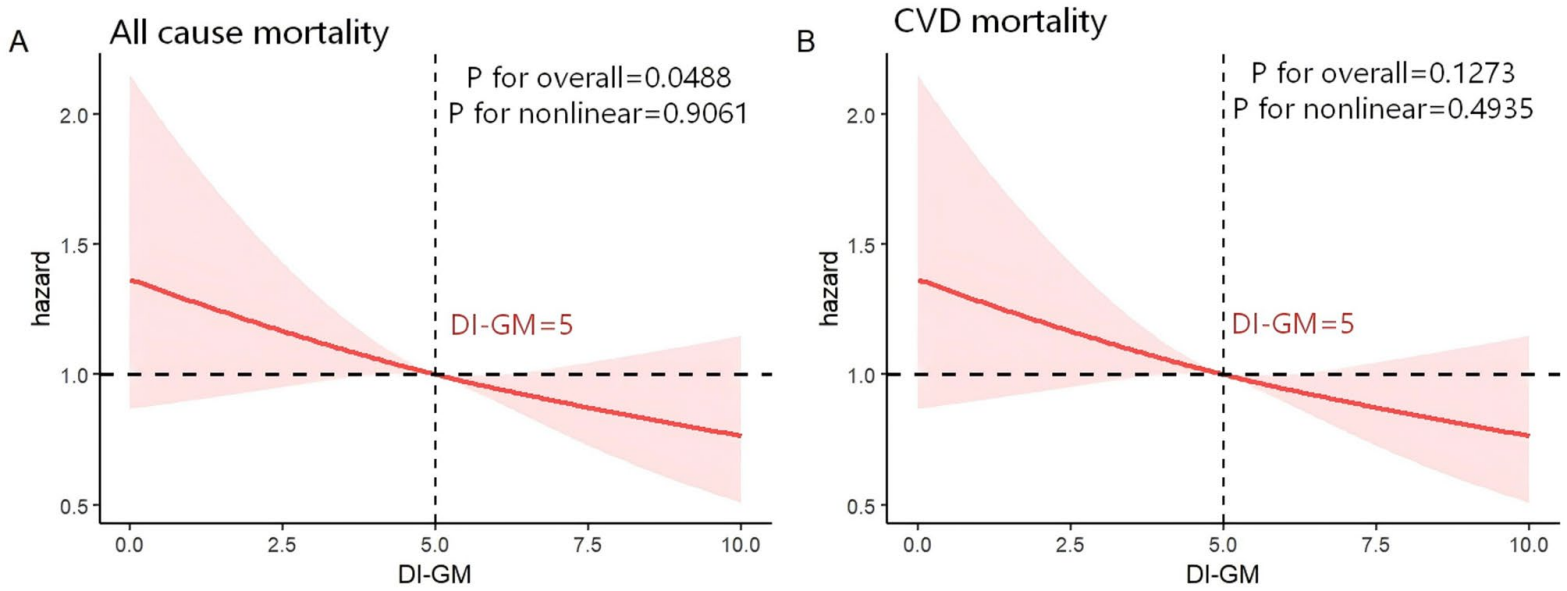
Association between DI-GM and all-cause and CVD mortality in hypertensive patients by RCS. (**A**) Linear association between DI-GM score and all-cause mortality. (**B**) Linear association between DI-GM score and CVD mortality. The model adjusted for sociodemographic characteristics (age, sex, and race), personal lifestyle and behavioral variables (education level, family size, insurance, marital status, smoking status, drinking status, BMI, sitting time, DASH), factors associated with health conditions (diabetes mellitus, hyperlipidemia, cancer, antidiabetic drugs, antihypertensive drugs, and lipid-lowering therapies). Abbreviations: DI-GM, dietary index for gut microbiota; CVD, cardiovascular disease; RCS, restricted cubic spline

**Fig. 3:**
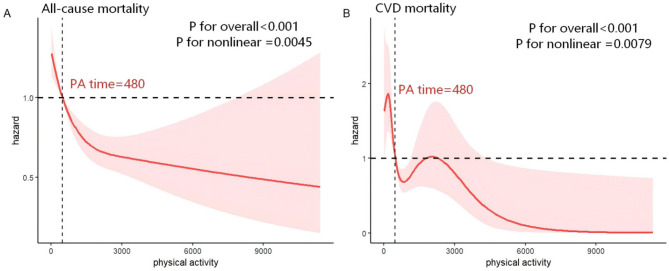
Association between PA time and all-cause and CVD mortality in hypertensive patients by RCS. (**A**) Linear association between PA time and all-cause mortality. (**B**) Linear association between PA time and CVD mortality. The model adjusted for sociodemographic characteristics (age, sex, and race), personal lifestyle and behavioral variables (education level, family size, insurance, marital status, smoking status, drinking status, BMI, sitting time, DASH), factors associated with health conditions (diabetes mellitus, hyperlipidemia, cancer, antidiabetic drugs, antihypertensive drugs, and lipid-lowering therapies)

Restricted cubic splines analysis was applied to explore the dose–response relationship between DI-GM scores and total physical activity time (minutes/week) with all-cause and CVD mortality among patients with hypertension, using three knots (10th, 50th, and 90th percentiles) for DI-GM with both all-cause and CVD mortality, three knots for total physical activity time with all-cause mortality, and five knots (5th, 25th, 50th, 75th and 90th percentiles) for total physical activity time with CVD mortality. After multivariable adjustment, higher DI-GM scores were associated with a reduced risk of all-cause mortality (Fig. 2A, P for overall = 0.0488), but showed no significant association with CVD mortality (Fig. 2B, P for overall = 0.1273). No evidence of linearity was observed for either all-cause or CVD mortality (P for non-linearity = 0.9061 and 0.4935, respectively). In contrast, longer total physical activity time was inversely related to both all-cause and CVD mortality (Fig. 3A, *P* < 0.001, P for non-linearity = 0.0045; Fig. 3B, *P* < 0.001, P for non-linearity = 0.0079).

### Interaction and joint analysis of DI-GM and PA patterns with mortality

**Fig. 4:**
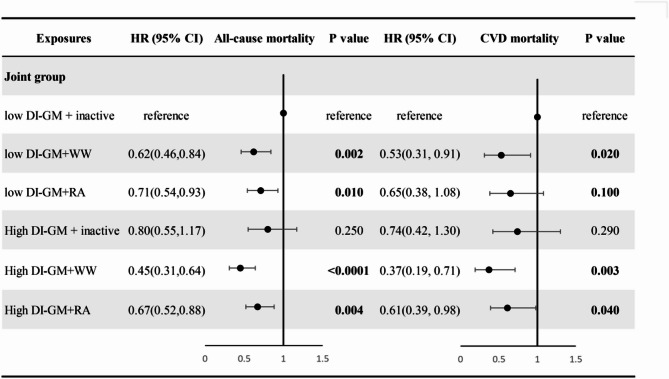
The forest plots show the joint association between DI-GM and PA patterns on all-cause and CVD mortality among 9082 hypertensive patients. The results were adjusted for sociodemographic characteristics (age, sex, and race), personal lifestyle and behavioral variables (education level, family size, insurance, marital status, smoking status, drinking status, BMI, sitting time, DASH), factors associated with health conditions (diabetes mellitus, hyperlipidemia, cancer, antidiabetic drugs, antihypertensive drugs, and lipid-lowering therapies). Abbreviations: DI-GM, dietary index for gut microbiota. PA patterns: inactive, weekend warrior, regularly active. WW, weekend warrior; RA, regularly active; High DI-GM: ≥6; Low DI-GM: <6

**Fig. 5:**
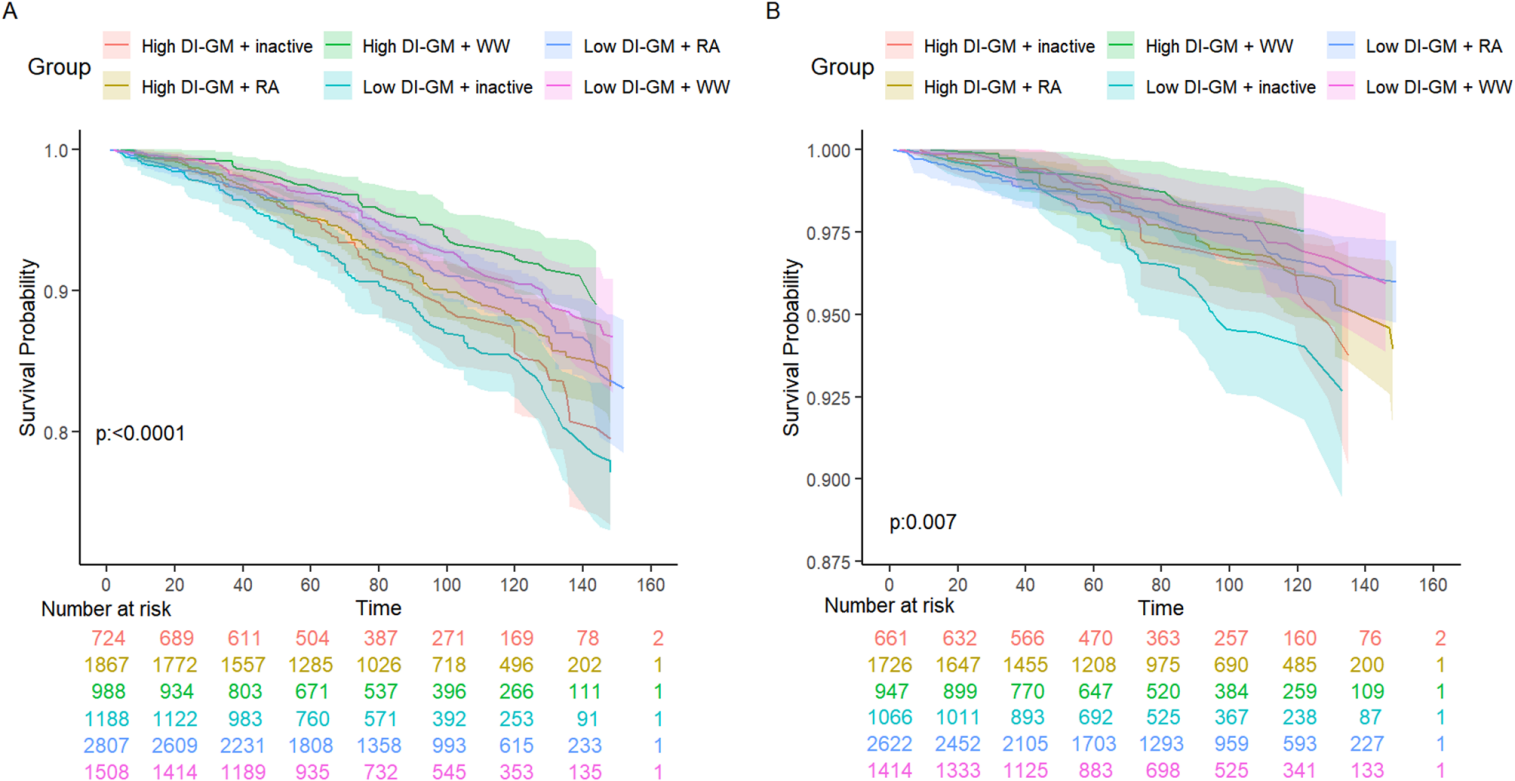
Kaplan-Meier curves show the all-cause and CVD survival probabilities of hypertensive patients with DI-GM levels and PA patterns. (**A**) all-cause survival with DI-GM and different patterns of PA; (**B**) CVD survival with DI-GM and different patterns of PA. The results were adjusted for sociodemographic characteristics (age, sex, and race), personal lifestyle and behavioral variables (education level, family size, insurance, marital status, smoking status, drinking status, BMI, sitting time, DASH), factors associated with health conditions (diabetes mellitus, hyperlipidemia, cancer, antidiabetic drugs, antihypertensive drugs, and lipid-lowering therapies)

We further assessed the interaction and joint association between the levels of DI-GM and the PA patterns with mortality (Supplementary Table 1). Compared with individuals who had low DI-GM and inactive PA patterns, the HR of all-cause mortality was 0.45 (95%CI, 0.31 to 0.64) for individuals who had high DI-GM levels and WW whereas the HR of all-cause mortality further decreased to 0.67 (95% CI, 0.52 to 0.88) in individuals who had high DI-GM levels and regularly active (Fig. 4). Compared to the low DI-GM and inactive group, the combination of low DI-GM and WW was significantly associated with reduced CVD mortality (HR = 0.53, 95% CI: 0.31–0.91, *P* = 0.02), the high DI-GM and WW group did not show a significant reduction in CVD mortality overall (HR = 0.37, 95% CI: 0.19–0.71, *P* = 0.003). In the interaction analysis (Table 4), regularly active physical activity showed both multiplicative (HR for interaction = 1.39, 95% CI: 1.02–1.90) and additive interaction with DI-GM on all-cause mortality in Model 1 (RERI = 0.28, 95% CI: 0.05–0.50). However, these associations were attenuated and became non-significant after further adjustment (Model 2 and Model 3). Overall, in the fully adjusted models, no evidence of multiplicative or additive interaction was observed between DI-GM and any physical activity patterns (regularly active or weekend warrior) in relation to either all-cause or CVD mortality. The KM curves revealed that hypertensive patients with high DI-GM and WW showed the significantly highest overall and CVD survival probability (Fig. 5).

**Table 4:** Interaction between DI-GM and WW with all-cause and CVD mortality in hypertensive patients in NHANES 2007–2018

		Model 1	Model 2	Model 3
**All-cause mortality**				
inactive vs. regularly active	Multiplicative interaction	**1.39 [1.02**,** 1.9]**	1.26 [0.92, 1.72]	1.21 [0.88, 1.66]
	Additive interaction (95% CI)			
	RERI	**0.28 [0.05**,** 0.5]**	0.22 [−0.01, 0.44]	0.18 [−0.05, 0.42]
inactive vs. weekend warrior	Multiplicative interaction	0.84 [0.56, 1.26]	0.79 [0.52, 1.18]	0.81[0.54,1.22]
	Additive interaction (95% CI)			
	RERI	0.04 [−0.21, 0.29]	0.04 [−0.2, 0.29]	0.001[−0.25,0.27]
**CVD mortality**				
inactive vs. regularly active	Multiplicative interaction	1.51 [0.85, 2.69]	1.34 [0.75, 2.38]	1.28 [0.72, 2.29]
	Additive interaction (95% CI)			
	RERI	0.34 [−0.06, 0.74]	0.26 [−0.13, 0.65]	0.22 [−0.19, 0.63]
inactive vs. weekend warrior	Multiplicative interaction	0.97 [0.45, 2.13]	0.92 [0.42, 2.01]	0.95 [0.43, 2.09]
	Additive interaction (95% CI)			
	RERI	0.15 [−0.27, 0.58]	0.18 [−0.21, 0.57]	0.14 [−0.3, 0.58]

Multiplicative interaction was evaluated using hazard ratios for the product term between the DI-GM levels (≥ 6 vs. <6) and PA patterns (inactive, weekend warrior, regularly active). DI-GM levels were coded as a binary variable (0 = < 6, 1 = ≥ 6), and PA patterns were dummy-coded with “inactive” as the reference category. Interaction terms were created by multiplying the binary DI-GM variable with each of the PA dummy variables. With “low DI-GM + inactive” set as the reference group. The multiplicative interaction term was constructed as the product of the binary indicators for DI-GM group and PA patterns. Statistical significance was indicated when the 95% confidence interval did not include 1. Additive interaction was evaluated using relative excess risk due to interaction (RERI) between the same DI-GM and PA categories, with the same reference group and coding, and was considered statistically significant when its 95% confidence interval did not include 0

### Subgroup and sensitivity analysis

Subgroup analyses stratified by age (< 45 vs. ≥45 years), sex (male vs. female), and sedentary time (< 240, 240–360, > 360 min/day) demonstrated that higher DI-GM scores, weekend warrior physical activity, and PAGM were consistently associated with lower risks of all-cause and CVD mortality. The inverse association between DI-GM and mortality was more pronounced among participants aged ≥ 45 years and appeared stronger in women than in men. Moreover, the joint protective effect of DI-GM and weekend warrior activity on all-cause mortality was particularly evident among individuals with longer sedentary time. (Supplementary Table 2).

In the sensitivity analysis, this association remained robust after excluding the participants who died within 24 months (Supplementary Table 3).

## Discussion

The findings of this population-based study indicated that (1) in patients with hypertension, DI-GM was associated with reduced all-cause mortality; (2) engaging in WW conferred comparable benefits for all-cause mortality to regular activity; and (3) no significant multiplicative or additive interactions were observed between DI-GM levels and WW with respect to all-cause and CVD mortality.

Our study found that DI-GM was inversely associated with all-cause mortality in hypertensive patients. Meanwhile, beneficial to gut microbiota was associated with a 5%–17% lower risk of all-cause mortality and a 4%–24% reduction in CVD mortality. The non-significant association between DI-GM and mortality in the crude model may be attributed to confounding factors, particularly age. With advancing age in hypertensive populations, the risk of mortality from cardiovascular and other diseases increases substantially [31]. We conducted age-stratified analyses, revealing a significant association in individuals aged ≥ 45 years; DI-GM was significantly associated with reduced risks of all-cause mortality. In middle-aged and older adults, gut microbiota dysbiosis is more prevalent [32]. However, specific microbiota alterations (e.g., enrichment of SCFA-producing bacteria) may exert protective effects on metabolic regulation, inflammatory responses, and immune function. These findings highlight the potential of DI-GM to enhance clinical outcomes in hypertensive patients, particularly among middle-aged and older adults. Within the NHANES database, the total DI-GM score ranges from 0 to 13. The median DI-GM score was 5.00 (IQR: 4.00 to 6.00). Specifically, the median score for dietary components unfavorable to gut microbiota was 3.00 (IQR: 2.00 to 3.00), whereas components beneficial to gut microbiota had a median score of 3.00 (IQR: 2.00 to 4.00). These findings indicated that DI-GM levels were generally low, particularly for dietary components beneficial to gut microbiota, which may neglect the beneficial role of diet and thus showed no association between the DI-GM diet and mortality. As a response to diet-induced changes in gut microbiota diversity, DI-GM reflects dietary patterns that are beneficial or harmful to gut health [33]. Changes in DI-GM indicate changes in dietary habits, which subsequently affect the diversity of the gut microbiota and are associated with the risk of mortality. For example, fermented dairy products are a unique beneficial component of DI-GM, containing beneficial microorganisms, microbial metabolites, and biological activities that shape the composition of the gut microbiota. Mazidi et al. [34] meta-analysis also showed that fermented dairy intake was associated with reduced all-cause mortality [34] and CVD mortality [35]. TMAO is a metabolite of the gut microbiome, derived primarily from choline (found in foods such as red meat, fish, poultry, and eggs) that is metabolized by the microbiome [36, 37] to produce trimethylamine, which is then produced by dry mycin monooxygenase 3 [36, 38]. A recent study by Heianza et al. [39] reported that participants with high TMAO levels had a 63% increased risk of all-cause mortality, independent of traditional risk factors such as smoking, obesity, and diabetes. These findings highlight the protective role of a high DI-GM pattern in reducing the risk of mortality in hypertensive patients.

Numerous studies have demonstrated a positive effect of PA on hypertension [40, 41], and several guidelines have suggested that patients with hypertension should increase their daily PA levels [42]. Our findings are largely in line with previous studies that have shown a correlation between increased levels of PA and a reduced risk of mortality [43, 44]. However, while earlier research had primarily focused on regular PA, our study expanded on this by exploring the associations of WW and mortality. Our study revealed that WW experienced a 30% reduction in all-cause mortality compared to inactive individuals, findings consistent with those reported by Mahe et al. [45]. However, our analysis indicated that the WW did not confer a protective effect on CVD mortality among hypertensive patients, contrasting with previous studies suggesting that WW reduce CVD mortality risks [46]. This discrepancy may be attributed to the intense bursts of high-intensity exercise characteristic of the WW, extensive research had shown that vigorous PA not only provides cardiovascular protection but is also associated with a higher incidence of acute cardiovascular events. Moreover, VPA may lead to negative cardiovascular adaptations, such as accelerated coronary artery calcification and increased release of cardiac biomarkers. Furthermore, our findings align with Huang et al.‘s previous work [47], which emphasized the broader benefits of WW for individuals who sit for extended periods. In the subgroup with sedentary time ≥ 360 min/d, patients had the lowest CVD mortality. It is crucial to note that sedentary time is independently associated with the risk of all-cause and CVD mortality, and WW should aim to minimize sedentary behavior on non-exercise days. However, it is indisputable that PA exerts a positive influence on the prognosis of hypertension. Therefore, we urge hypertensive patients to maintain regular exercise to support the management and prevention of the condition.

To our knowledge, this is the first study to evaluate the combined effect of DI-GM and WW on mortality in hypertensive patients. Previous studies had investigated the independent effects of diet and PA on people with hypertension, and rarely considered their combined effects. The combined analysis allowed us to explore the unique and combined contribution of each factor to mortality outcomes, providing more comprehensive disease management for patients with hypertension. The joint analysis showed that high levels of DI-GM combined with WW or regular PA significantly reduced the risk of all-cause mortality or CVD mortality in hypertensive patients. It was associated with an approximately 16–52% lower risk of all-cause mortality in hypertensive patients. This finding suggests that dietary or exercise interventions alone may not be sufficient to fully address mortality in hypertensive patients and that synergy between the two may be key and consistent with findings from previous evidence. More recently, results from 346,627 participants in the UK Biobank [48] showed that the category with the highest quartile of PA participation and the highest diet quality score was associated with lower all-cause mortality, CVD mortality, and cancer mortality. In the few studies that examined the joint association of PA and diet with cardiovascular outcomes, greater reductions in CVD events [49] and risk of CVD mortality [50] were reported when higher levels of PA were combined with a better Mediterranean diet or better diet quality. Therefore, WW with high blood pressure should strictly adhere to several favorable dietary factors, such as increasing the consumption of fiber, fermented dairy products, and whole grains, while reducing the consumption of processed meat, fat intake, and refined grains.

The association between DI-GM, WW, and mortality risk in hypertensive patients may be partially explained by several potential mechanisms. First, DI-GM is linked to gut microbiota diversity and may enhance the production of substances such as SCFAs, SCFAs can exert anti-inflammatory effects by inhibiting the expression of inflammatory cells and can participate in gluconeogenesis and stimulate the sympathetic nervous system [51]. Meanwhile, engaging in WW exercise regimens confers similar benefits to regularly active, mitigating atherosclerosis and thereby delaying the progression of hypertension [52]. MVPA can increase the diversity of intestinal flora; specifically, MPA and VPA were associated with a higher abundance of the butyrate-producers Faecalibacterium prausnitzii and Roseburia spp [53]. Of note, DI-GM and WW might share similar biological pathways to influence the prognosis of hypertensive patients, which could explain the joint effect of these two factors. An alternative explanation for our findings is that the observed benefits may be attributable to the nutritional composition of the diet itself, rather than exclusively mediated through gut microbiota–related pathways. Recent cohort studies have shown that frequent intake of fermented dairy products is associated with lower risk of incident hypertension and favorable lipid profiles, even after adjusting for BMI and other lifestyle factors [54]. Likewise, higher intake of dietary fiber has been repeatedly linked to reduced cardiovascular disease risk, with ~ 7% risk reduction per 10 g/day increase, through improvements in blood pressure, systemic inflammation, and lipid metabolism, independent of microbiome changes [55, 56]. These findings suggest that while gut microbiota modulation is a plausible mechanism, part of the cardiometabolic benefits may also derive from the intrinsic effects of fermented dairy and fiber themselves, underscoring the need for future studies to disentangle these pathways using both microbial and metabolic biomarkers.

Our study has several strengths. First, we are the first to investigate the combined effects of DI-GM and WW on mortality outcomes in hypertensive patients. Second, we employed sample weighting and multivariate adjustment in all Cox regression models to enhance the reliability and robustness of our findings. Finally, we also conducted a series of sensitive analyses to validate the robustness of the findings and identify high-risk subgroups.

Nevertheless, we acknowledge some limitations. First, PA and diet data were largely self-reported, so recall bias is inevitable. For DI-GM, we excluded individuals who participated in only one 24-hour diet recall to improve our effectiveness. Second, the study only measured DI-GM and PA at baseline and did not collect information on the dynamics of these factors during follow-up. Therefore, further prospective cohort studies or randomized controlled trials are needed for further analysis.

## Conclusions

In conclusion, DI-GM was negatively associated with mortality risk in hypertensive patients, and engaging in WW confers similar benefits for all-cause mortality as regular exercise. In the joint analysis, WW was consistently linked to a lower risk of all-cause mortality, regardless of dietary habits. These findings supported the integration of combined dietary and PA interventions into comprehensive hypertension management strategies.

## Supplementary Information

Supplementary Material 1

## Data Availability

The National Health and Nutrition Examination Survey (NHANES) data are publicly available at https://www.cdc.gov/nchs/nhanes/index.htm.
