# Supplementary material for: Joint association of dietary index for gut microbiota and weekend warrior physical activity pattern with mortality among hypertensive patients
Source: Nutr Metab (Lond). 2025 Nov 27;23:1. doi: 10.1186/s12986-025-01026-8 (PMC12763907; doi:10.1186/s12986-025-01026-8)
Supplement: Supplementary file 1 — Supplementary Material 1 [file 12986_2025_1026_MOESM1_ESM.docx]

Table 1 Association of DI-GM and PA patterns with mortality in hypertensive patients in NHANES 2007 – 2018

| Mortality outcome | Death/No. | Mode l | | Model 2 | | Model 3 | |
| --- | --- | --- | --- | --- | --- | --- | --- |
|  |  | HR (95% CI) | *P* | HR (95% CI) | *P* | HR (95% CI) | *P* |
| All-cause mortality |  |  |  |  |  |  |  |
| low DI-GM + inactive |  | reference | reference | reference | reference | reference | reference |
| low DI-GM+WW | 124/1508 | 0.54(0.39,0.73) | **<0.0001** | 0.55(0.40,0.76) | **<0.001** | 0.62(0.46,0.84) | **0.002** |
| low DI-GM+RA | 270/2807 | 0.66(0.51,0.87) | **0.003** | 0.68(0.51,0.89) | **0.01** | 0.71(0.54,0.93) | **0.01** |
| High DI-GM + inactive | 88/724 | 0.85(0.60,1.21) | 0.37 | 0.80(0.56,1.16) | 0.24 | 0.80(0.55,1.17) | 0.25 |
| High DI-GM+WW | 56/988 | 0.43(0.30,0.62) | **<0.0001** | 0.40(0.28,0.58) | **<0.0001** | 0.45(0.31,0.64) | **<0.0001** |
| High DI-GM+RA | 207/1867 | 0.73(0.56,0.96) | **0.02** | 0.66(0.50,0.87) | **0.004** | 0.67(0.52,0.88) | **0.004** |
| CVD mortality |  |  |  |  |  |  |  |
| low DI-GM + inactive | 52/1066 | reference | reference | reference | reference | reference | reference |
| low DI-GM+WW | 30/1414 | 0.46(0.27,0.80) | **0.01** | 0.45(0.26, 0.78) | **0.005** | 0.53(0.31, 0.91) | **0.02** |
| low DI-GM+RA | 85/2622 | 0.59(0.36,0.97) | **0.04** | 0.59(0.36, 0.97) | **0.04** | 0.65(0.38, 1.08) | 0.1 |
| High DI-GM + inactive | 25/661 | 0.72(0.43,1.20) | 0.21 | 0.68(0.40, 1.14) | 0.14 | 0.74(0.42, 1.30) | 0.29 |
| High DI-GM+WW | 15/947 | 0.36(0.20,0.66) | **<0.001** | 0.32(0.17, 0.57) | **<0.001** | 0.37(0.19, 0.71) | **0.003** |
| High DI-GM+RA | 66/1726 | 0.67(0.43,1.04) | 0.08 | 0.57(0.37, 0.89) | **0.01** | 0.61(0.39, 0.98) | **0.04** |

Note: Model 1 served as the unadjusted analysis;

Model 2: further adjusted for sociodemographic characteristics (age, sex, and race);

Model 3: further adjusted for personal lifestyle and behavioral variables (education level, family size, insurance, marital status, smoking status, drinking status, BMI, sitting time, DASH ), factors associated with health conditions (diabetes mellitus, hyperlipidemia, cancer, antidiabetic drugs, antihypertensive drugs, and lipid-lowering therapies).

Abbreviations: DI-GM, dietary index for gut microbiota. PA patterns: inactive, weekend warrior, regularly active. WW, weekend warrior; RA, regularly active; High DI-GM: ≥6; Low DI-GM: <6.

Table 2 Subgroup analysis shows the robust association between the joint association of DI-GM and PA patterns with the all-cause and CVD mortality among hypertensive patients in NHANES 2007–2018.

|  | age | | sex | | sitting time | | |
| --- | --- | --- | --- | --- | --- | --- | --- |
|  | <45 | >=45 | Male | Female | <4 | 4~6 | >6 |
| **All-cause mortality** |  |  |  |  |  |  |  |
| DI-GM | 1.01(0.84, 1.22) | **0.94(0.89,0.99)** | 0.97(0.92,1.04) | **0.88(0.80,0.96)** | 1.01(0.93,1.09) | 0.92(0.83,1.02) | 0.93(0.86,1.02) |
| Unfavorable to gut microbiota | 0.99(0.72, 1.37) | 1.10(0.99,1.21) | 1.16(1.04,1.29) | 0.97(0.80,1.16) | 1.11(0.94,1.30) | 1.11(0.94,1.31) | 1.05(0.88,1.26) |
| Beneficial to gut microbiota | 1.00(0.80, 1.25) | **0.88(0.82,0.94)** | **0.89(0.81,0.98)** | **0.86(0.78,0.94)** | 0.95(0.85,1.07) | **0.86(0.77,0.96)** | **0.89(0.81,0.97)** |
| DI-GM |  |  |  |  |  |  |  |
| 0-3 | reference | reference | reference | reference | reference | reference | reference |
| 4 | 0.93(0.35, 2.44) | 0.86(0.64,1.14) | 0.89(0.63,1.26) | 0.74(0.48,1.15) | 0.78(0.46,1.32) | 0.70(0.44,1.11) | 1.23(0.76,2.00) |
| 5 | 0.93(0.43, 2.00) | 0.82(0.64,1.05) | 0.93(0.68,1.26) | 0.61(0.40,0.92) | 0.95(0.56,1.63) | 0.61(0.38,0.96) | 1.09(0.67,1.79) |
| ≥6 | 0.84(0.33, 2.16) | 0.73(0.58,0.92) | 0.82(0.60,1.12) | 0.56(0.37,0.84) | 0.86(0.61,1.23) | 0.65(0.42,1.02) | 0.82(0.52,1.31) |
| PA patterns |  |  |  |  |  |  |  |
| Inactive | reference | reference | reference | reference | reference | reference | reference |
| WW | 0.79(0.28, 2.23) | **0.57(0.45,0.73)** | **0.57(0.44,0.75)** | **0.60(0.40,0.89)** | 0.62(0.38,1.02) | **0.63(0.41,0.96)** | **0.62(0.42,0.90)** |
| RA | 0.96(0.41, 2.26) | 0.75(0.62,0.90) | 0.69(0.54,0.90) | 0.83(0.63,1.07) | 0.83(0.57,1.19) | 0.93(0.67,1.28) | 0.57(0.41,0.81) |
| PAGM |  |  |  |  |  |  |  |
| low DI-GM + inactive | reference | reference | reference | reference | reference | reference | reference |
| low DI-GM+WW | 0.64(0.22, 1.90) | 0.62(0.45,0.85) | 0.66(0.47,0.93) | 0.65(0.40,1.07) | 0.80(0.41,1.55) | 0.84(0.49,1.41) | 0.49(0.33,0.74) |
| low DI-GM+RA | 0.95(0.38, 2.38) | 0.69(0.51,0.94) | 0.80(0.56,1.15) | 0.60(0.40,0.91) | 0.83(0.48,1.43) | 0.94(0.61,1.46) | 0.53(0.34,0.82) |
| High DI-GM + inactive | 0.73(0.08, 6.47) | 0.80(0.54,1.17) | 1.19(0.75,1.91) | 0.56(0.31,1.02) | 1.12(0.46,2.69) | 1.07(0.62,1.82) | 0.60(0.35,1.04) |
| High DI-GM+WW | 0.88(0.18, 4.24) | 0.42(0.30,0.59) | 0.56(0.37,0.83) | 0.26(0.14,0.49) | 0.49(0.22,1.08) | 0.43(0.23,0.81) | 0.52(0.30,0.92) |
| High DI-GM+RA | 0.76(0.24, 2.39) | 0.66(0.51,0.86) | 0.67(0.47,0.95) | 0.67(0.45,1.01) | 0.91(0.54,1.54) | 0.96(0.63,1.48) | 0.41(0.25,0.69) |
| **CVD mortality** |  |  |  |  |  |  |  |
| DI-GM | 0.80( 0.39, 1.63) | 0.92(0.84,1.01) | 0.96(0.85, 1.09) | 0.80(0.68, 0.93) | 1.05(0.87, 1.26) | 0.89(0.74, 1.08) | 0.93(0.80, 1.07) |
| Unfavorable to gut microbiota | 1.22( 0.51, 2.91) | 1.09(0.93,1.26) | 1.10(0.93, 1.31) | 1.03(0.71, 1.50) | 1.17(0.90, 1.52) | 1.10(0.84, 1.44) | 1.15(0.90, 1.46) |
| Beneficial to gut microbiota | 0.66( 0.29, 1.49) | 0.86(0.77,0.96) | 0.90(0.78, 1.04) | 0.73(0.60, 0.88) | 1.00(0.80, 1.24) | 0.82(0.68, 0.99) | 0.83(0.69, 0.99) |
| DI-GM |  |  |  |  |  |  |  |
| 0-3 | reference | reference | reference | reference | reference | reference | reference |
| 4 | 0.30( 0.03, 3.46) | 0.72(0.45,1.13) | 0.78(0.42, 1.45) | 0.45(0.22, 0.88) | 0.86(0.30, 2.50) | 0.48(0.23, 0.98) | 0.96(0.42, 2.18) |
| 5 | 0.46( 0.03, 7.11) | 0.67(0.38,1.18) | 0.85(0.41, 1.77) | 0.35(0.16, 0.76) | 0.78(0.34, 1.78) | 0.42(0.17, 1.06) | 1.17(0.48, 2.85) |
| ≥6 | 0.24( 0.01, 9.67) | 0.63(0.43,0.94) | 0.76(0.42, 1.38) | 0.33(0.18, 0.62) | 0.92(0.45, 1.88) | 0.49(0.24, 1.01) | 0.76(0.35, 1.63) |
| PA patterns |  |  |  |  |  |  |  |
| Inactive | reference | reference | reference | reference | reference | reference | reference |
| WW | 0.55( 0.14, 2.11) | 0.51(0.32,0.81) | 0.46(0.26, 0.84) | 0.59(0.30, 1.16) | 0.62(0.20, 1.88) | 0.61(0.29, 1.29) | 0.41(0.21, 0.81) |
| RA | 0.54( 0.08, 3.42) | 0.71(0.49,1.02) | 0.66(0.40, 1.11) | 0.75(0.49, 1.14) | 0.54(0.27, 1.08) | 0.98(0.57, 1.69) | 0.58(0.31, 1.05) |
| PAGM |  |  |  |  |  |  |  |
| low DI-GM + inactive | reference | reference | reference | reference | reference | reference | reference |
| low DI-GM+WW | 0.19( 0.01, 2.55) | 0.51(0.27,0.97) | 0.49(0.24, 1.02) | 0.65(0.29, 1.46) | 0.64(0.21, 2.02) | 0.78(0.29, 2.06) | 0.26(0.13, 0.54) |
| low DI-GM+RA | 0.42( 0.05, 3.59) | 0.64(0.35,1.16) | 0.64(0.31, 1.35) | 0.63(0.32, 1.22) | 0.29(0.12, 0.68) | 0.85(0.38, 1.92) | 0.71(0.30, 1.67) |
| High DI-GM + inactive | 0.00( 0.00, 0.00) | 0.81(0.43,1.51) | 0.88(0.42, 1.82) | 0.57(0.25, 1.34) | 0.51(0.11, 2.38) | 0.79(0.29, 2.17) | 0.79(0.41, 1.51) |
| High DI-GM+WW | 0.93( 0.05, 16.00) | 0.36(0.18,0.72) | 0.39(0.16, 0.97) | 0.26(0.10, 0.71) | 0.32(0.07, 1.49) | 0.34(0.11, 1.05) | 0.50(0.17, 1.43) |
| High DI-GM+RA | 0.20( 0.00, 11.02) | 0.67(0.40,1.13) | 0.63(0.34, 1.16) | 0.54(0.29, 0.99) | 0.65(0.27, 1.58) | 0.97(0.46, 2.04) | 0.32(0.15, 0.69) |

The model adjusted for sociodemographic characteristics (age, sex, and race), personal lifestyle and behavioral variables (education level, family size, insurance, marital status, smoking status, drinking status, BMI, sitting time, DASH ), factors associated with health conditions (diabetes mellitus, hyperlipidemia, cancer, antidiabetic drugs, antihypertensive drugs, and lipid-lowering therapies).

Table 3 Sensitive analysis shows the robust association between the joint association of DI-GM and PA patterns with the all-cause and CVD mortality among hypertensive patients in NHANES 2007–2018. (Excluding the participants who died within 24 months).

| Mortality outcome | Mode l | | Model 2 | | Model 3 | |
| --- | --- | --- | --- | --- | --- | --- |
|  | HR (95% CI) | *P* | HR (95% CI) | *P* | HR (95% CI) | *P* |
| All-cause mortality |  |  |  |  |  |  |
| low DI-GM + inactive | reference | reference | reference | reference | reference | reference |
| low DI-GM+WW | 0.57(0.42,0.78) | **<0.001** | 0.58(0.42,0.81) | **0.001** | 0.65(0.48,0.88) | **0.01** |
| low DI-GM+RA | 0.63(0.49,0.81) | **<0.001** | 0.64(0.49,0.83) | **<0.001** | 0.66(0.51,0.87) | **0.003** |
| High DI-GM+inactive | 0.92(0.64,1.33) | 0.67 | 0.87(0.59,1.27) | 0.46 | 0.85(0.58,1.26) | 0.43 |
| High DI-GM+WW | 0.45(0.31,0.64) | **<0.0001** | 0.41(0.29,0.59) | **<0.0001** | 0.46(0.33,0.65) | **<0.0001** |
| High DI-GM+RA | 0.76(0.58,1.00) | **0.05** | 0.68(0.52,0.90) | **0.01** | 0.68(0.52,0.90) | **0.01** |
| CVD mortality |  |  |  |  |  |  |
| low DI-GM + inactive | reference | reference | reference | reference | reference | reference |
| low DI-GM+WW | 0.49(0.28,0.85) | **0.01** | 0.47(0.27, 0.82) | **0.01** | 0.55(0.32, 0.95) | **0.03** |
| low DI-GM+RA | 0.46(0.28,0.75) | **0.002** | 0.46(0.28, 0.74) | **0.002** | 0.49(0.30, 0.81) | **0.01** |
| High DI-GM+inactive | 0.73(0.43,1.23) | 0.24 | 0.68(0.40, 1.17) | 0.17 | 0.72(0.40, 1.30) | 0.28 |
| High DI-GM+WW | 0.38(0.21,0.70) | **0.002** | 0.33(0.18, 0.60) | **<0.001** | 0.39(0.20, 0.74) | **0.004** |
| High DI-GM+RA | 0.65(0.41,1.05) | **0.08** | 0.55(0.34, 0.89) | **0.01** | 0.58(0.36, 0.93) | **0.03** |

Note: Model 1 served as the unadjusted analysis;

Model 2: further adjusted for sociodemographic characteristics (age, sex, and race);

Model 3: further adjusted for personal lifestyle and behavioral variables (education level, family size, insurance, marital status, smoking status, drinking status, BMI, sitting time, DASH ), factors associated with health conditions (diabetes mellitus, hyperlipidemia, cancer, antidiabetic drugs, antihypertensive drugs, and lipid-lowering therapies).

Abbreviations: DI-GM, dietary index for gut microbiota. PA patterns: inactive, weekend warrior, regularly active. WW, weekend warrior; RA, regularly active; High DI-GM: ≥6; Low DI-GM: <6.
